# Anatomical and ontogenetic influences on muscle density

**DOI:** 10.1038/s41598-021-81489-w

**Published:** 2021-01-22

**Authors:** Kaitlyn C. Leonard, Nikole Worden, Marissa L. Boettcher, Edwin Dickinson, Kailey M. Omstead, Anne M. Burrows, Adam Hartstone-Rose

**Affiliations:** 1 Department of Biological Science, North Carolina State University, 3546 Thomas Hall, 112 Derieux Place, Raleigh, NC 27607 USA; 2 Department of Biological Sciences, Meredith College, 3800 Hillsborough St, Raleigh, NC 27607 USA; 3 College of Medicine, Medical University of South Carolina, 96 Jonathan Lucas St. Ste. 601, MSC 617, Charleston, SC 29425 USA; 4 Department of Physical Therapy, Duquesne University, 600 Forbes Ave., Pittsburgh, PA 15282 USA

**Keywords:** Musculoskeletal models, Animal physiology

## Abstract

Physiological cross-sectional area (PCSA), an important biomechanical variable, is an estimate of a muscle’s contractile force potential and is derived from dividing muscle mass by the product of a muscle’s average fascicle length and a theoretical constant representing the density of mammalian skeletal muscle. This density constant is usually taken from experimental studies of small samples of several model taxa using tissues collected predominantly from the lower limbs of adult animals. The generalized application of this constant to broader analyses of mammalian myology assumes that muscle density (1) is consistent across anatomical regions and (2) is unaffected by the aging process. To investigate the validity of these assumptions, we studied muscles of rabbits (*Oryctolagus cuniculus*) in the largest sample heretofore investigated explicitly for these variables, and we did so from numerous anatomical regions and from three different age-cohorts. Differences in muscle density and histology as a consequence of age and anatomical region were evaluated using Tukey’s HSD tests. Overall, we observed that older individuals tend to have denser muscles than younger individuals. Our findings also demonstrated significant differences in muscle density between anatomic regions within the older cohorts, though none in the youngest cohort. Approximately 50% of the variation in muscle density can be explained histologically by the average muscle fiber area and the average percent fiber area. That is, muscles with larger average fiber areas and a higher proportion of fiber area tend to be denser. Importantly, using the age and region dependent measurements of muscle density that we provide may increase the accuracy of PCSA estimations. Although we found statistically significant differences related to ontogeny and anatomical region, if density cannot be measured directly, the specific values presented herein should be used to improve accuracy. If a single muscle density constant that has been better validated than the ones presented in the previous literature is preferred, then 1.0558 and 1.0502 g/cm^3^ would be reasonable constants to use across all adult and juvenile muscles respectively.

## Introduction

Physiological cross-sectional area (PCSA) is an architectural property of muscle that directly relates to force production capabilities—muscles with higher PCSAs can produce proportionally more force than muscles with lower PCSAs. As such, PCSA has been frequently used to contextualize dietary^1–11^ and locomotor adaptations^12–16^ across taxa. Unlike muscle mass and fascicle length, which are measured directly, PCSA is derived: calculated as a function of muscle mass, average fascicle length and muscle density^17^. The constants used for muscle density (~ 1.06 g/cm^3^) are commonly taken from several different studies^18–20^. These sources share some commonalities: their model specimens were all adult individuals and their samples were taken from similar anatomical regions. Therefore, the use of these constants makes the assumption that muscle density is static regardless of age and anatomical region—an assumption that this study aims to address.

Gersh et al.^18^ used mature guinea pigs as their model system and were analyzing the specific gravity or relative density of skeletal muscle due to changes in pressure. The authors did not specify the anatomical region from which they took their tissue sample, but they found no significant differences in the specific density of the muscle after decompression. Mendez and Keys^20^ used muscles of the lower limb and “sometimes” include the psoas muscle of mature rabbits and dogs to evaluate muscle density. Though they specify the muscles they utilized which include the quadriceps cruralis, gastrocnemius, and tibialis, the authors do not specify what muscles and how many of each were included in each of the 13 muscle samples for rabbits and 12 muscle samples for dogs^20^. Lastly, Murphy and Beardsley^19^ evaluated the mechanical properties of the soleus muscle of adult cats (n = 6) and as an aside measured density because it was necessary to calculate PCSA. The conclusions of these studies^18–20^ all converged upon very similar densities (1.065, 1.0597, and 1.0564 g/cm^3^, respectively). This convergence may reflect similarities in study design between these experiments, which were all comprised exclusively of adult individuals, and limited variation among anatomical regions. Therefore, given these similarities, using a constant for muscle density makes some inherent assumptions.

### Assumption 1

The first assumption made seemingly universally by functional morphologists that is important to evaluate is that muscle density remains relatively constant throughout an individual’s life. For this assumption to be true, muscle mass and muscle volume would have to change proportionally because density is defined as the mass per unit of volume. However, given that other architectural properties of muscle have been demonstrated to be dynamic and change throughout the lifespan of animals (see, for example^21–23^), this is also likely to be the case for muscle density. For example, muscle mass has been shown to decline with increasing age—a condition that has been termed as sarcopenia^24,25^. Other previous studies conducted provide more direct evidence that muscle density is variable with age^26–28^. For instance, Imamura et al.^27^ investigated the size and density of human sacrospinalis and psoas major muscles with respect to age using computed tomography and observed an increase in density until middle-age with a subsequent decline. While the authors noted that the differences they observed were statistically significant, they did not specify what these differences were. Additional evidence suggesting muscle density changes with age is provided by a study conducted by Newton et al.^28^ who observed a decline in the density of the masseter and medial pterygoid muscles with advancing age. Overarching trends within this literature suggest that muscle density will increase until approximately middle-age and decline throughout senescence.

In addition to these studies that suggest muscles will vary based on their gross characteristics (e.g., mass), it is also likely that they will change microscopically and in composition (especially during growth), resulting in changes in muscle density. Muscles grow three different ways which include increasing the number of muscle fibers, increasing the size of the muscle fibers, and lastly, increasing the length of the muscle fibers^29^. Skeletal muscle is predominantly comprised of muscle fibers with connective tissue such as collagen and fat dispersed throughout^30^. Therefore, as muscle grows it is likely that the proportions of the microscopic components will change resulting in differences in muscle density.

### Assumption 2

It is additionally assumed in most functional myology studies that muscle density is not influenced by the anatomical region from which the samples are taken. The study conducted by Méndez and Keys^20^ used lower limb muscles including the quadriceps cruralis, gastrocnemius, tibialis, and occasionally included the hip flexor psoas, while the other most commonly cited reference for muscle density, the study by Murphy and Beardsley^19^, only evaluated the soleus. This is potentially problematic as the density constant determined by these authors is used to make determinations about muscles from all anatomical regions and it has been clearly demonstrated that muscles are variable in composition. For instance, Faucitano et al.^31^ found that fat content within individual muscle fascicles can vary throughout the same muscle. Muscles that are higher in fat (i.e., greater degree of “marbling”—as the food industry calls it) should have a lower density relative to leaner muscles because fat has a significantly lower density of 0.936 g/cm^3^^32^. Additionally, a generalized inverse trend has been demonstrated within muscles between fat content and water content^33–35^. This variability in muscle composition will presumably be reflected in muscle density. Fat content within skeletal muscle has been demonstrated to be correlated with its microscopic organization. For instance, Kauffman and Safanie noted that organized, but widely dispersed fasciculi correlated with high lipid content^36^. The present study plans to address this by sampling a certain area of a cross-section of each muscle to determine the percent of this area comprised of muscle fibers. A higher percentage of muscle fibers in theory should be correlated to less fat content and ultimately greater density.

In addition to variable fat content, it has been reported that collagen—the most substantial constituent element of connective tissue within skeletal muscle-can comprise between 3 and 30% of a muscle’s total protein^37^. Therefore, muscles with a lot of connective tissue will likely be denser. A good example of this would be the masseter because it is a complex muscle consisting of several fascial layers with connective tissue throughout.

### Predictions

In effort to address these assumptions and based on previous literature we predict the following:

1. Based on previous studies^26,27^, we predict that muscle density will vary based on age within our sample—increasing until prime adulthood.
2. Furthermore, we anticipate that this increase in density will be histologically correlated with an increase in the size of the muscle fibers. We expect this to influence density because larger fibers will inherently contain more proteins which are denser than water resulting in a slightly higher density.

## Materials and methods

To test these hypotheses a sample (n = 66) of New Zealand white *Oryctolagus cuniculus* rabbit cadavers was obtained from a commercial meat farm, Brittany Ridge Farms. All animals were euthanized according to USDA standards prior to obtaining them and were therefore deemed “exempt” by NC State IACUC. The specimens were subdivided into three age-cohorts which consisted of individuals approximately 3 weeks (n = 18), 8 months (n = 30) and 2 years of age (n = 18; Table 1). These age-cohorts were selected based on the life history of this breed of rabbit and availability. (As commercial breeders of rabbits for the food industry do not keep animals beyond prime breeding age, we were not able to study the effects of senescence in this species, which lives to 7 years old^38^; see “Limitations and future directions” below). Three-week old rabbits were chosen to represent the truly juvenile cohort as they do not wean until approximately 30 days of age^39,40^. Sexual maturity is achieved by 6 months of age^41^, therefore the 8-month old rabbits represent sexually mature individuals. By the age of 2 years (the oldest age-cohort that we could obtain from the commercial farm) rabbits have reached full adult size.

**Table 1:** *Oryctolagus cuniculus* sample demographics.

Cohort	Age	Males	Females	Total
C1	3-weeks old	7	11	18
C2	8-months old	11	19	30
C3	2-years old	10	8	18

In order to evaluate the effects of anatomical region on muscle density, muscles from four different regions were selected, including the head, forelimb, hindlimb and the trunk. Individual muscles were chosen as representative of these regions and also relatively diverse in subjective myological properties (e.g., muscles with a lot of connective tissue, “lean” muscles, and states in between), and included the masseter, digastric, quad labii, extensor carpi radialis longus (ECRL), pronator teres (PT), gastrocnemius, soleus, plantaris and psoas minor. After excision, muscles were weighed to the nearest 0.0001 g using a Mettler Toledo New Classic (MS-105) analytical balance and the density of each muscle at 20.0˚C was determined using a Mettler Toledo density kit (MS-DNY-54), which automatically calculates density using Archimedes principle. The kit requires first weighing the sample in air and then in an auxiliary liquid, for which we chose deionized water.

**Figure 1:**
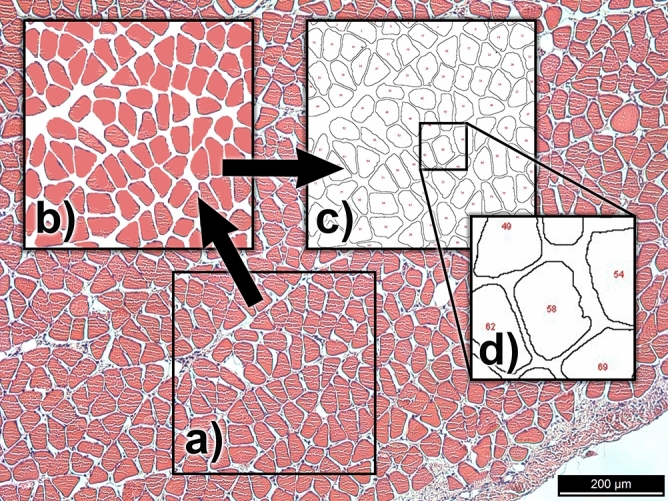
Stages of the histological image processing: (**a**) raw image obtained of an 8-month old rabbit soleus (Specimen ID: A54_Soleus) muscle, (**b**) 400 × 400 μm section preprocessed in Photoshop to simplify for measurement, (**c**) outline schematic produced by using the “analyze particles” function in ImageJ, (**d**) partial fibers are excluded by number.

Additionally, in an effort to better account for differences in muscle composition, a subsample of muscles was histologically evaluated (Fig. 1). To do this, three sections from each from each of the nine sampled muscles from two rabbits from each cohort (one male and one female from the two younger cohorts and two males from the older cohort) were cross-sectioned perpendicular to their fibers. For muscles that have multiple components in which the fascicular orientation varies throughout (e.g., masseter), the section was taken perpendicular to the most superficial fascicles. We then embedded these samples in paraffin, sectioned them at 10–12 μm and then stained using hematoxylin and eosin. The slides were photographed using a Leica (CTR5500) microscope and three 400 μm by 400 μm sections were sampled (chosen to minimize major vessels, nerves and tendons) using Photoshop (CC2019). These sections were then processed further using ImageJ (IJ1.46r) (Fig. 1). After the scale was set appropriately, the images were binarized, and then any holes within the individual fibers were filled using the “fill holes” tool. The “Analyze particles” function was then used to collect information about the area of each muscle fiber and the percent of each sampled section that consisted of fiber area. To determine the average fiber area, any partial fibers that may have been captured within our sampled section were excluded (e.g., on the perimeter of the frame). The percent fiber area was calculated by summing all of the fiber areas for each of the three sampled sections and dividing by the total sampled area.

All statistical analyses were conducted using JMP Pro14 (SAS). Tukey's HSD tests (essentially an all-pairs t-tests; alpha = 0.05) were conducted to compare the mean densities between age-cohorts and anatomical regions. Tukey's HSD tests were conducted to identify not only the presence of significant differences in mean density but to determine where the significances lie. This test was used to compare density across the anatomical regions of each age-cohort as well as across the age-cohorts for each muscle. Further Tukey’s HSD tests were conducted to evaluate to determine if average fiber area and percent of the sampled area that was comprised of muscle fibers differed significantly between age-cohorts. To evaluate the scaling relationship between muscle density and average fiber area and percent fiber area reduced major axis (RMA) linear regressions were conducted. This type of analysis accounts for error in both the x and y axes^42^ and is commonly employed when evaluating scaling relationships between variables.

### Ethics approval and consent to participate

The use of cadaveric materials is exempt from North Carolina State University’s IACUC.

### Consent for publication

All authors listed on this publication provide their consent for the work to be published.

## Results

**Figure 2:**
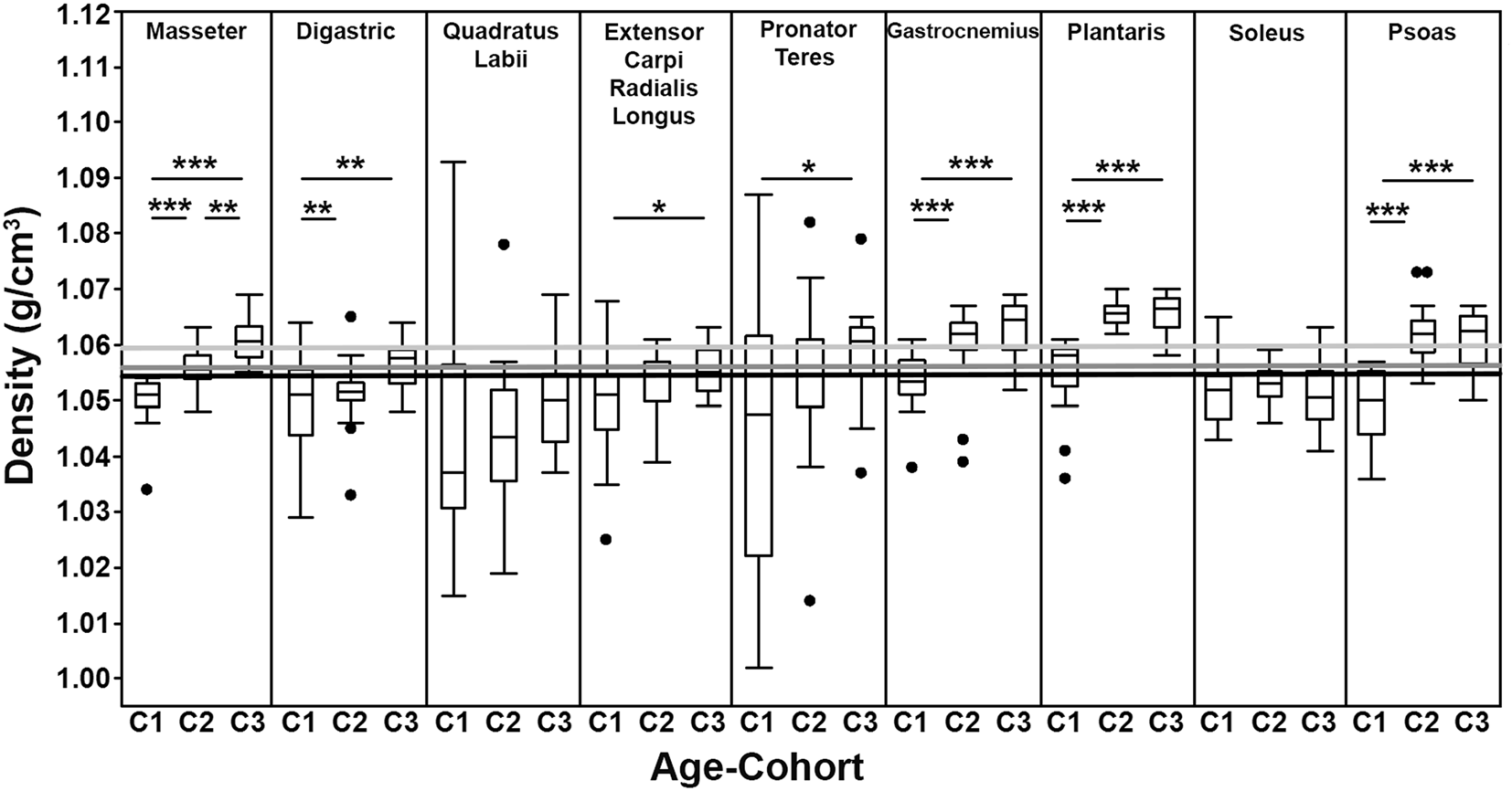
The distributions for the density of each muscle per each of the three cohorts: C1 = 3 weeks; C2 = 8 months; C3 = 2 years old. The lines above the box plots indicate significant differences between connected columns (* p < 0.05, ** p < 0.01, *** p < 0.0001) for each muscle. The light gray and medium gray lines depict the constants taken from Mendez and Keys (1.0597 g/cm^3^; 1960) and Murphy and Beardsley (1.0564 g/cm^3^; 1974) respectively. The black line represents the grand mean of all the muscles measured in this study (1.0546 g/cm^3^; n = 594 muscles). One data outlier (C1 of the quadratus labii; density of 1.16 g/cm^3^) omitted for graphical clarity.

The distributions of muscle density for the three age-cohorts and each muscle indicates variation based on both age and anatomical region (Fig. 2). The quadratus labii was consistently the least dense while the plantaris was the densest (Table 2). The mean density of all muscles except the soleus became greater with increasing age (Table 2; Fig. 2) though, because of variance, not all of these increases across the full sample of muscles were significant.

**Table 2:** The mean muscle density, mean standard error and results within each age-cohort (C1 = 3 weeks old; C2 = 8 months old; C3 = 2 years old).

Muscle	Age-cohort	Mean muscle density (g/cm^3^)	Mean standard error
Masseter	C1	1.0501	0.0011
	C2	1.0558	0.0007
	C3	1.0606	0.0009
Digastric	C1	1.0497	0.0020
	C2	1.0513	0.0009
	C3	1.0565	0.0010
Quadratus Labii	C1	1.0482	0.0079
	C2	1.0432	0.0022
	C3	1.0499	0.0019
Extensor Carpi Radialis Longus	C1	1.0494	0.0025
	C2	1.0529	0.0011
	C3	1.0556	0.0009
Pronator Teres	C1	1.0453	0.0060
	C2	1.0542	0.0022
	C3	1.0589	0.0021
Gastrocnemius	C1	1.0534	0.0013
	C2	1.0606	0.0011
	C3	1.0630	0.0011
Plantaris	C1	1.0551	0.0016
	C2	1.0653	0.0003
	C3	1.0656	0.0008
Soleus	C1	1.0514	0.0014
	C2	1.0529	0.0007
	C3	1.0508	0.0013
Psoas	C1	1.0493	0.0015
	C2	1.0616	0.0009
	C3	1.0611	0.0013
All regions	C1	1.0502	0.0012
	C2	1.0553	0.0006
	C3	1.0580	0.0006

The mean density of the masseter increased with increasing age and the masseter is the only muscle that differed significantly between each age-cohort (Table 2). A similar trend was observed when all muscles for each age-cohort were analyzed collectively (C1 n = 162; C2 n = 270; C3 n = 162; Table 2). Contrastingly the quadratus labii and the soleus were the only two muscles that did not show any significant differences in muscle density between the three age-cohorts (Table 2). The digastric, gastrocnemius, plantaris and psoas followed similar trends to one another. The youngest individuals had muscle densities that were significantly different than those of the older two cohorts, but the average densities between the older two cohorts did not differ from one another (Table 2). The extensor carpi radialis longus (ECRL) and pronator teres both exhibited the same trend. The average muscle density within the 3-week age-group was not significantly different than the 8-month olds but was significantly different than the 2-year olds. The specimens from the older two cohorts were also not significantly different than one another (Table 2).

Within the 3-week old cohort there were no significant density differences between any muscles; however, this was not the case within the other two age-cohorts (Table 3).

**Table 3:** “Connected Letter” report of Tukey's HSD results of density differences between the anatomical regions of each age-cohort (C1 = 3 weeks old; C2 = 8 months old; C3 = 2 years old).

Muscle	Tukey's HSD results across C1	Tukey's HSD results across C2	Tukey's HSD results across C3
Masseter	A	BCD	ABC
Digastric	A	BCD	BCD
Quadratus Labii	A	E	D
Extensor Carpi Radialis Longus	A	D	CD
Pronator teres	A	CD	ABC
Gastrocnemius	A	ABC	AB
Plantaris	A	A	A
Soleus	A	D	D
Psoas	A	AB	ABC

Muscles connected by letters cannot be statistically separated at α = 0.05. For example, the density of gastrocnemius, plantaris and psoas cannot be distinguished across any of the three age-cohorts (all connected by the letter “A”, as are all of the muscles of the density indistinct youngest cohort), but the density of soleus is significantly different (connected to other muscles labeled “D”, but without a connection to “A”) than that of gastrocnemius in the two older cohorts.

The most consistent region-specific variation observed between the 8-month and 2-year old cohorts occurred within the triceps surae, with the plantaris being significantly denser than the gastrocnemius and soleus muscles (Table 3). There were no significant differences observed in muscle density between the pronator teres and ECRL muscles in either of the latter two age-cohorts (Table 3).

**Figure 3:**
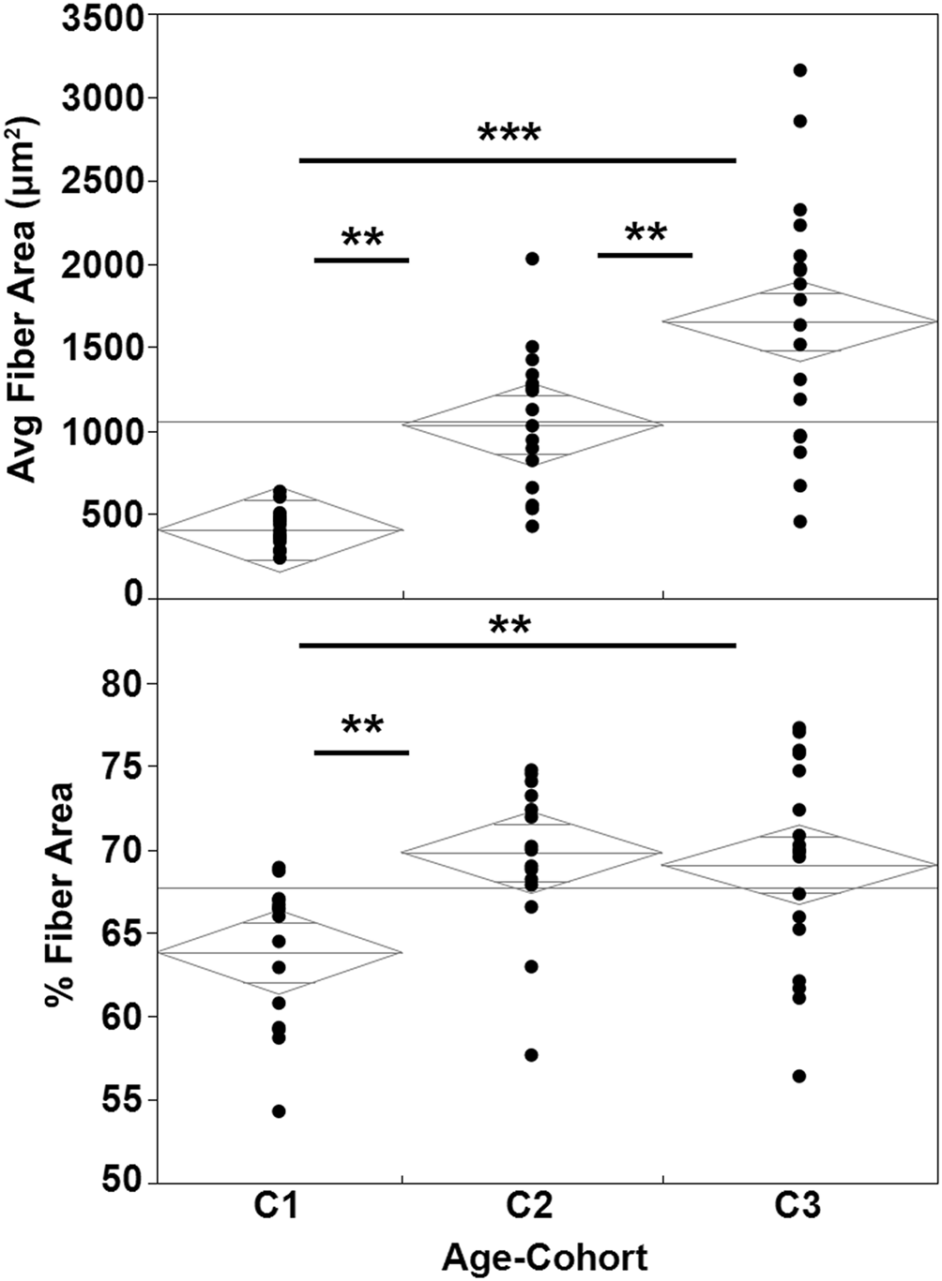
Distributions and mean diamonds for the histologically measured average fiber area and percent fiber area for each of the muscles (n = 18 muscles from 2 individuals) measured in each of the three cohorts (C1 = 3 weeks old; C2 = 8 months old; C3 = 2 years old). The lines above indicate significant differences between connected columns (*p < 0.01, **p < 0.001, ***p < 0.0001).

When comparing the distributions of the histologically measured average fiber areas between each age-cohort, (Fig. 3; Table 4), average fiber area was significantly higher in the 8-month old cohort than the 3-week old cohort and also significantly higher in the 2-year old cohort than the 8-month old cohort. We also compared the percent of the sampled histological area that was occupied by muscle fibers (Fig. 3; Table 5) and we observed statistically significant lower percent area occupied by muscle fibers in the 3-week cohort than either of the older cohorts. However, the average percent fiber area of the sample area was not significantly different between the 8-month and 2-year old cohorts.

**Table 4:** Average fiber area (μm^2^), standard deviation (μm^2^) and standard error (μm^2^) for each muscle of a subset of each age-cohort (C1 = 3 weeks old; C2 = 8 months old; C3 = 2 years old) and the connecting letter report from a Tukey’s HSD test comparing the three groups.

Age-cohort	Average fiber area (μm^2^)	Std. dev. (μm^2^)	Std. error mean (μm^2^)
C1	393.87	129.48	31.40
C2	1037.65	427.68	103.73
C3	1657.02	736.32	173.55

**Table 5:** Average percent fiber area, standard deviation and standard error for each muscle for a subset of each age-cohort (C1 = 3 weeks old; C2 = 8 months old; C3 = 2 years old) and the connecting letter report from a Tukey's HSD test comparing the three groups.

Age-cohort	Average % fiber area	Std. dev	Std. error mean
C1	62.40	7.22	1.75
C2	69.82	4.43	1.07
C3	69.08	6.06	1.43

**Figure 4:**
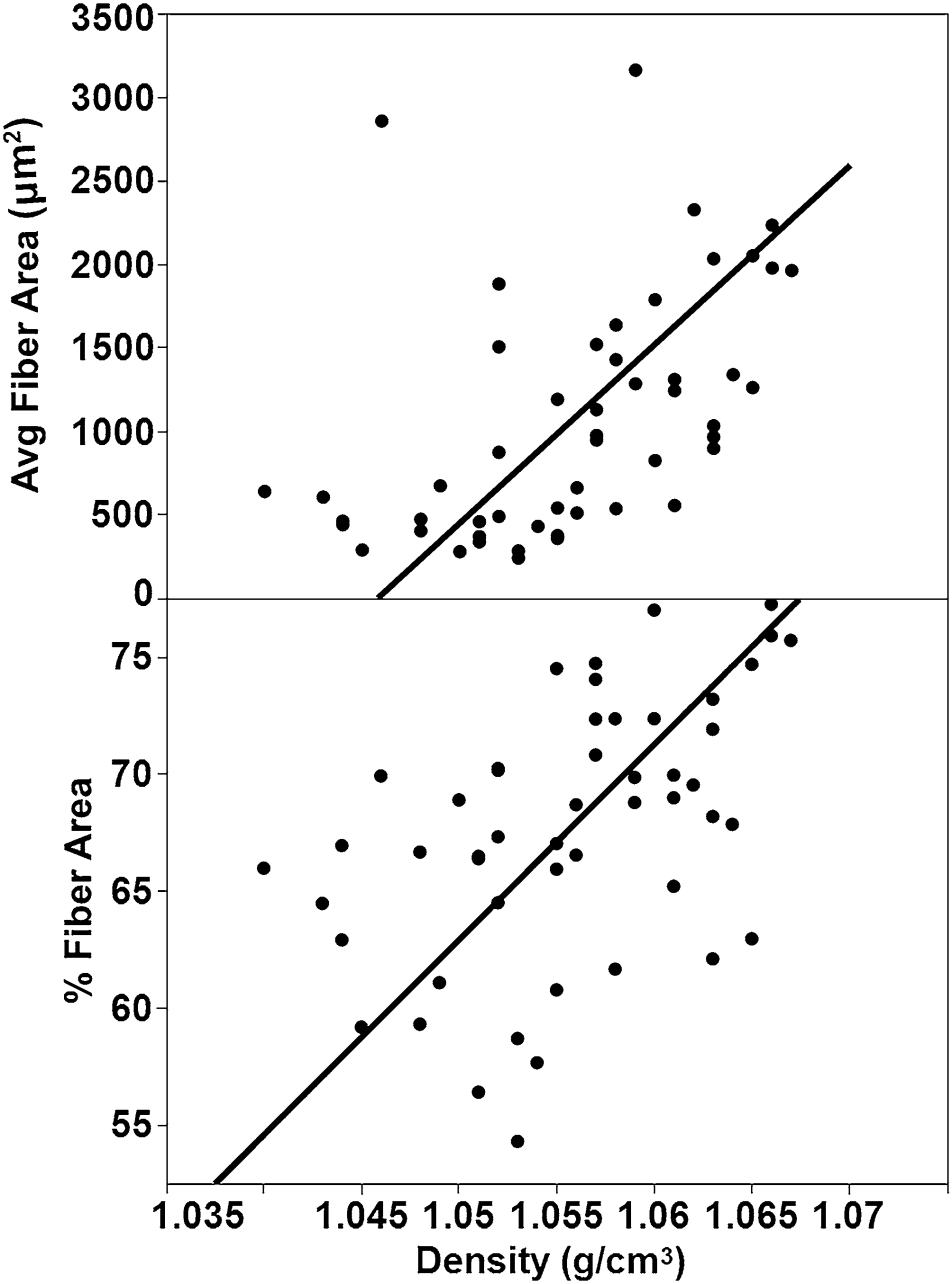
Reduced major axis regressions of average fiber area (top; r^2^ = 0.49) and % fiber area against (bottom; r^2^ = 0.49) density combined for all muscles and cohorts.

To evaluate the relationship between muscle density and average fiber area and average percent fiber area we conducted a reduced major axis regression (Fig. 4). We observed that approximately 49% of the variation observed in muscle density is explained by the average fiber area and average percent fiber area—with individuals with denser muscles having larger fiber areas (i.e., bigger cross-sectional areas of the fibers) and the sample regions consisting of a greater proportion of muscle fibers within the sampled regions (i.e., higher percent of the sampled region was made up of muscle fibers; Fig. 4).

## Discussion

Accurate representation of muscle density is crucial when calculating PCSA, an important biomechanical variable that is a direct correlate of muscle force production potential. However, the current practice in the field of substituting a value for muscle density as universally applicable from previous studies that included very conscribed samples^18–20^, obscures the variability in this value that relates to ontogeny and anatomical region. To address this, this study comprehensively evaluated variation in muscle density in different age groups and parts of the body and sought to explain these differences histologically.

As we predicted based on the findings of Imamura et al.^27^ consisting of increasing muscle density within the sacrospinalis and the psoas major muscles until middle-age in humans, we also observed the general trend that for each muscle we evaluated, the average density was successively higher within the latter age-cohorts (Fig. 2, Table 2). However, these differences were not always significant between the age-cohorts for each muscle—suggesting differential trends in development based on the functional demands throughout the animal’s life history. More specifically, while the mean density of each muscle (except soleus) increased in each age-cohort, those increases were only significant for about half of the cohort to cohort comparisons (Fig. 2). Although soleus had a relatively tight range of density measurements and did not exhibit this ontogenetic trend, the other muscles for which this trend was least significant (i.e., quadratus labii and, to a lesser extent, extensor carpi radialis longus and pronator teres) were the muscles that had the widest standard deviations in muscle density. As the means of these densities increased like those of the other muscles, though with these notable wider standard deviations, if the sample sizes were increased, the ontogenetic trend in these muscles might also reach statistical significance.

The fact that soleus does not change density across the age-cohorts is rather surprising. Like the other two muscles of the triceps surae, it has fairly little variation in muscle density—though the mean densities, especially of the two older cohorts are notably lower than those of the gastrocnemius and plantaris. From a functional perspective, it is unclear why this muscle’s density would scale differently (or, as it were, not at all) over ontogeny, though perhaps it is because of functional/activation differences in these three crural muscles.

There do not appear to be clear regional differences in muscle density; the forelimb muscles are not generally more or less dense than the hindlimb muscles, nor (with the exception of the relatively light quadratus labii) are the head muscles or the trunk muscle (psoas) significantly notable. Thus, while there are significant differences in specific muscles in adult rabbits, these seem to fall *within* anatomical regions and are not general trends *between* them. This suggests that there may be important functional differences in synergistic muscles within the same system (e.g., the triceps surae) that influence their density as there is no apparently homogeneity in these anatomical muscle groups.

The differences that we observed in density generally increasing with age were, at least in part, explained by their histology: the average fiber area also increased with age. We also found that the percent of the sampled area that consisted of muscle fibers increased significantly between the 3-week old cohort and the 8-month cohort and the 3-week old and 2-year old cohorts but did not differ significantly between the 8-month old and 2-year old cohort.

Overall, our findings demonstrate significant differences in muscle density occurring both across ontogeny and anatomical region. Although we observed no statistically significant regional differences in muscle density within 3-week old rabbits, significant trends were observed within the two older cohorts. The lack of significant differences observed in youngest cohort could have two contrasting explanations. The first is that, the musculature has not developed enough to reflect the diversity of their inherent anatomical properties. The second plausible explanation is that muscles of the juveniles even within the same region could vary too widely—perhaps because of important differences in maturation at this age—such that the Tukey's HSD test would not be able to detect more subtle differences.

While the findings of our study demonstrate statistically significant differences in muscle density based on ontogeny and anatomical region, the practical significance of this may be relatively small: Inter-muscular differences appear rather subtle—meaning that if it is *not feasible* to directly measure muscle density, the use of a “constant” is reasonable. In other words, although there are significant differences, the magnitude of those differences authors should not worry excessively about these differences if density cannot be measured on a muscle by muscle basis. However, the use of the previously determined constants that were derived from a small subset of muscles from adult individuals (i.e., those used in^19,20^) should no longer be considered the best practice; this study presents a variety of values for muscle density (Table 6) that could be applied to more specific analyses based on age and structural characteristics of the muscle. For example, if a researcher is studying the temporalis muscle—a complex muscle consisting of several constituent layers and substantial fascial sheets, substituting a value for density that was taken from the masseter would at least provide some structural similarity. Another example, for a specimen of an unknown age, a density value of 1.0546 g/cm^3^ (Fig. 2) may be more appropriate as it was derived from a large sample (n = 594 muscles) of muscles from individuals of various ages, spanning greater anatomical breadth than the previously used constants. Researchers could also select other tailored constants if the muscle of a similarly aged-cohort has been incorporated here. For instance, densities of 1.0558 g/cm^3^ and 1.0502 g/cm^3^ would be appropriate for general adult and juvenile muscles respectively; and 1.0549 g/cm^3^ and 1.0474 g/cm^3^ for adult and juvenile for *forelimb* mm; 1.0597 g/cm^3^ and 1.0533 g/cm^3^ for adult and juvenile *leg* mm.; and 1.0518 g/cm^3^ and 1.0493 g/cm^3^ for adult and for juvenile *head* mm.

**Table 6:** Average muscle density by structural characteristics and age.

Muscle	Structural description	Age-cohort	Average muscle density (g/cm^3^)
Masseter	Robust and complex containing multiple fascial layers	C1	1.0501
		C2	1.0558
		C3	1.0606
Digastric	Cylindrical with a distinct distal tendon	C1	1.0497
		C2	1.0513
		C3	1.0565
Quadratus Labii	Thin and highly associated with the skin and connective tissue	C1	1.0482
		C2	1.0432
		C3	1.0499
Extensor Carpi Radialis Longus	Strap-like muscle with a distinct distal tendon	C1	1.0494
		C2	1.0529
		C3	1.0556
Pronator Teres	Strap-like muscle with a highly integrated and inseparable tendon	C1	1.0453
		C2	1.0542
		C3	1.0589
Gastrocnemius	Consists of a lateral and medial head that converge at a central tendinous sheet	C1	1.0534
		C2	1.0606
		C3	1.0630
Plantaris	Fusiform shaped with a substantial distal tendon	C1	1.0551
		C2	1.0653
		C3	1.0656
Soleus	Cylindrical with a distinct distal tendon	C1	1.0514
		C2	1.0529
		C3	1.0508
Psoas minor	Thin and fragile–lacking substantial sheets of connective tissue	C1	1.0493
		C2	1.0616
		C3	1.0611

### Limitations and future directions

This study produced significant findings; however, it is not without limitations. For example, because histological samples are, by their nature generally relatively small, we were not able to evaluate what are likely more subtle significant differences in average fiber area and percent fiber area between the older two cohorts.

While our study found significant differences histologically in the average fiber area and percent fiber area between age-cohorts we were still not able to explain other important elements of the tissue in a comprehensively quantitative manner. For instance we were able to make some inferences about how an increase in fiber area may be driving an increase in muscle density with age (i.e., larger muscle fiber areas thereby an increase in the amount of protein present), but were not able to quantify the proportion of other types of tissues present that may also be contributing to these differences (i.e., fat and collagen etc.). Future studies could incorporate a trichrome stain into the methods—allowing for the differentiation of these tissues which could then be quantified using the methods developed within this study.

Another histological element that is not incorporated here that may prove useful to incorporate into future studies is immunohistochemical fiber typing. For example, fiber type composition has been demonstrated to differ between the gastrocnemius and soleus muscles with the soleus muscle containing a greater proportion of Type I or slow twitch fibers^43^. Fiber-type analyses may help to explain why within the latter two age-cohorts the plantaris and gastrocnemius were consistently denser than the soleus muscle. This might also help to elucidate the similarities in density like, for example, was observed between the ECRL and the PT despite functional and structural differences. Future studies could further evaluate the correlation between fiber-type and the aging condition to elucidate the relationships between fiber-type, age and the dynamic functional demands of life.

Another limitation to this study is that our sample did not include an age-cohort that was a true representation of senescence. We procured our highly controlled sample from a commercial meat farm where they do not have animals that reach senescence because they would no longer be reproductively efficient. In a future extension of this work, it would be valuable to find and incorporate some data from senescent individuals perhaps from show rabbit breeders or to replicate this density research on a taxon for which a broader age range is available (e.g., the mouse lemurs used in^22,23^, though a larger taxon would probably yield more accurately measured densities). Additionally, further studies are needed to determine the species-specificity of these values of muscle density presented here.

Lastly, while the current study examines the relationship between muscle density in relation to ontogeny and anatomical region, future studies should examine it in relation to other conditions including use/disuse and disease—factors that clearly could affect the concentration of muscle proteins and therefore the relationship between PCSA and density.

## Conclusions

The implications of this study are great in that it is the first study to explicitly evaluate the density of muscles across broad anatomical regions and ages—while the highly cited standard values for muscle density were collected on limited samples as part of studies that were not explicitly trying to establish a constant that has become so broadly used. Ultimately this study has demonstrated the variability in muscle density associated with ontogeny and anatomy. We have also provided a variety of values for specific muscles if colleagues happen to be studying the same or similar muscles that we evaluated, and more general values that can be used more generically (namely a value of 1.0558 g/cm^3^ for adult muscles, 1.0502 g/cm^3^ for juveniles, 1.0597 g/cm^3^ for adult legs and 1.0518 g/cm^3^for adult head muscles) if muscle density cannot be directly measured. In short, if possible, researchers should measure muscle density directly. However, if this is not possible, the more specific density values presented herein can help improve the accuracy of architectural analysis. More work needs to be done to elucidate how these values change across species and how factors such as specimen handling and fluid preservation and future studies on the effects of how senescence and muscle fiber type may also influence muscle density.

## Data Availability

Data can be made available by contacting Dr. Adam Hartstone-Rose.
